# Economic burden of cancer in India: Evidence from cross-sectional nationally representative household survey, 2014

**DOI:** 10.1371/journal.pone.0193320

**Published:** 2018-02-26

**Authors:** Sunil Rajpal, Abhishek Kumar, William Joe

**Affiliations:** Institute of Economic Growth, Delhi University Enclave, North Campus, Delhi, India; Universita degli Studi di Firenze, ITALY

## Abstract

With the ongoing demographic and epidemiological transition, cancer is emerging as a major public health concern in India. This paper uses nationally representative household survey to examine the overall prevalence and economic burden of cancer in India. The age-standardized prevalence of cancer is estimated to be 97 per 100,000 persons with greater prevalence in urban areas. The evidence suggests that cancer prevalence is highest among the elderly and also among females in the reproductive age groups. Cancer displays a significant socioeconomic gradient even after adjusting for age-sex specifics and clustering in a multilevel regression framework. We find that out of pocket expenditure on cancer treatment is among the highest for any ailment. The average out of pocket spending on inpatient care in private facilities is about three-times that of public facilities. Furthermore, treatment for about 40 percent of cancer hospitalization cases is financed mainly through borrowings, sale of assets and contributions from friends and relatives. Also, over 60 percent of the households who seek care from the private sector incur out of pocket expenditure in excess of 20 percent of their annual per capita household expenditure. Given the catastrophic implications, this study calls for a disease-based approach towards financing such high-cost ailment. It is suggested that universal cancer care insurance should be envisaged and combined with existing accident and life insurance policies for the poorer sections in India. In concluding, we call for policies to improve cancer survivorship through effective prevention and early detection. In particular, greater public health investments in infrastructure, human resources and quality of care deserve priority attention.

## Introduction

The term “Cancer” is derived from the Greek word “*Karkinos”* (for crab) which refers to a generic non-communicable disease (NCD) characterized by growth of malignant (cancerous or neo-plasms) abnormal cells (tumor/lump) in any part of the human body [1–2]. Although several forms of cancer have been detected, the most common sites of these tumors in human bodies are lungs, stomach, colorectal, liver, and breasts [3–5]. Globally, the cancer etiology as well as epidemiology has received significant attention of researchers and policymakers [6–12]. In fact, cancer is the second leading cause of deaths worldwide and accounts for a share of 13 percent in total global deaths (or 8.7 million deaths) [13–14]. The prevalence of cancer was conventionally much evident in developed nations, but in recent years, it has increased substantially in developing countries as well. The estimates from Global Burden of Disease (GBD)suggest that about 70 percent of all cancer deaths are now concentrated among low- and middle-income countries [15]. However, cancer research and treatment are one of the most challenging fields in biomedical sciences and oncologists have been struggling to ensure greater survival chances among cancer patients. In general, there is a consensus that about 60 percent of cancer deaths can be prevented with improved preventive (removing the causes of disease so theta exposure to risk is minimal) and screening (test or procedure used to detect disease) facilities [16–17]. Given the fact that much of the cancer survival is associated with early diagnosis, access to state-of-the-art medical technology is a prominent policy concern for low-and middle-income countries. The problem increases manifold for developing nations such as India that has poor geographical coverage of medical services and negligible financial protection in health.

Against this backdrop, this paper examines the distributional patterns in self-reported prevalence and economic burden of cancer in India. According to WHO, India has a cancer mortality rate of 79 per 100,000 deaths and accounts for over 6 percent of total deaths [18]. These numbers are very close to those of high-income countries. Further, the cancer mortality in India is projected to increase to over 900,000 deaths by the end of this decade [19]. Also, with higher burden of breast and uterine cancer, the cancer incidence in India is also identified with a significant gender dimension [20–22]. Most importantly, in India, and as elsewhere, the term cancer resonates shock and fear because of two concurrent reasons; first, very high treatment costs and second, poor chances of survival [23]. The financial burden associated with cancer treatment can force patients and households to acute misery and even insolvency [24–26]. Some of the earlier hospital-based studies find that, on average, a household spends about Rs. 36,812 for the entire cancer therapy excluding non-medical costs [24]. It is also noted that out of pocket (OOP) expenditure on cancer hospitalization is about 2.5 times of overall average hospitalization expenditure [27]. While catastrophic expenditure on cancer inpatient treatment is highest among all NCDs, poor health financing mechanisms and heavy reliance on out-of-pocket healthcare payments compels several cancer patients to resort to distressed means for treatment financing [28–30]. In fact, previous studies on India suggest that about 60 and 32 percent households resort to borrowings and contributions (from friends and relatives) respectively for cancer hospitalization [27].

Although, there are a few small-areas or hospital-based studies that highlight the concern of high OOP expenditures but certainly these are insufficient to comprehend the situation from a macro-perspective [31–36]. For example, Mohanti et al (2011) in their study presented average expenditure estimates on cancer inpatient care from a public hospital in the national capital, Delhi. Another study by Swaminathan et al. (2009) focuses on the association between education and cancer prevalence in South India and observes greater prevalence of cancer among less educated men and women. However, most of the studies investigating OOP expenditure and its catastrophic consequences have not approached the concern from a disease perspective [25, 37]. Besides, most of these hospital-based evidences in India have focused on specific forms of cancer (like breast cancer, colorectal cancer. liver cancer) and do not present comprehensive understanding of socioeconomic patterns and distributions of OOP expenditure on cancer treatment [38–42].

A disease-centric approach on cancer assumes salience because of specific national policy commitments to ensure universal access to health care at affordable prices [43]. Although, provisioning and access to cancer treatment has been an important item on the public health agenda but the policy intent cannot proceed very far without understanding the socioeconomic patterns in cancer prevalence, treatment-seeking and financing. In fact, there is limited evidence to inform policymaking regarding socioeconomic dimension of the disease which can further disallow discussions on health financing mechanisms. Therefore, with this motivation, we analyze the nationally representative household health and health care survey data to examine the economic burden of cancer on Indian households. A specific focus is on describing the broad patterns in catastrophic out of pocket expenditures and distressed financing incurred by households. These findings are further discussed to arrive at policy alternatives to approach this grave public health concern.

## Data and methods

### Data

This study is based on nationally representative data from *Social Consumption*: *Health* survey (71^st^ round) of India. The survey was conducted in 2014 by National Sample Survey Organization (NSSO), Ministry of Statistics and Program Implementation, Government of India. A key objective of *Social Consumption*: *Health* survey is to obtain data on aspects of morbidity, treatment-seeking and financing of hospitalization (inpatient) and ambulatory (outpatient) care services for the reference period of 365 days and 15 days respectively. The ailments for which such medical care is sought, the extent of use of Government hospitals, and the expenditure incurred on treatment received from public and private sectors, is also available through this survey. Additionally, the survey provides household level information on demographics and access to services and utilities as well as individual level data on age, sex, education, monthly per capita expenditure and primary occupation of households.

### Survey design

The *Social Consumption*: *Health* Survey interviews are conducted with a representative sample of households randomly selected through a stratified multi-stage survey design covering India. A rural/urban stratification is created within clusters called state-regions, which comprises of a continuous group of districts within a State or union territory having similar characteristics. Within each district of a State/Union Territory, two strata were formed: the rural stratum comprising of all rural areas in the district, and the urban stratum comprising of all urban areas in the district. Selection of first stage units is based on the principle of probability proportional to size with circular systematic sampling of census-identified villages in the rural sector and urban frame survey blocks in the urban sector of each district. Larger sample villages and blocks are divided into a suitable number of "hamlet-groups"/"sub-blocks" of roughly equal population content. Second-stage sampling constituted the households belonging to only two of these hamlet-groups, selected circular systematically in case of sample villages, and one randomly selected sub-block in the case of sample blocks. Households within a village are categorized in two strata based on affluence. From these strata, households are circular systematically selected to constitute the final sample. This cross-sectional survey data was collected during January to June 2014. The 71^st^ round of Morbidity and Healthcare Survey covers a sample of 65,932 households and 335,499 individuals.

### Outcomes

First, we present the self-reported prevalence of cancer across socioeconomic groups. For analytical purposes, prevalence of cancer refers to any person suffering from any type of cancer or received cancer inpatient or outpatient treatment (or both). These estimates are presented along with the percentage of cancer patients undergoing treatment in public and private healthcare facilities separately.

Further, the estimates for average out of pocket (OOP) medical and total expenditure on cancer inpatient care across SES groups are also reported. The medical expenditure mainly includes information on doctor’s/surgeon’s fee, expenditure on medicines, diagnostic tests, bed charges and other miscellaneous expenses (like attendant charges, physiotherapy charges, personal medical appliances, blood and oxygen). The total expenditure is the summation of medical expenditure and transport charges for patient, food transport on others, expenditure on escorts and their lodging charges. It is observed that the number of sample cases for cancer outpatient care is almost negligible whereas most of the cancer patients have reported receipt of hospitalization care. Therefore, the expenditure analysis specifically focuses on hospitalization expenditure related to cancer treatment across socioeconomic groups.

A high reliance on OOP spending is a major concern and hence it can potentially jeopardize the customary living standards of the households [44–45]. To unravel such concerns, we present an analysis of incidence of catastrophic expenditure by examining proportion of households that incur greater OOP expenditure as a share of their household expenditure. We employ conventional expenditure thresholds of 10, 20 and 40 percent of gross annual per capita household expenditure to discern the magnitude and socioeconomic patterns of such catastrophic expenditure related to cancer inpatient treatment. Furthermore, we also investigate percentage of households largely relying on distress financing mechanisms to receive cancer treatment. The survey elicits information regarding the major source of financing to capture whether bulk of the out of pocket expenditure was incurred via distressed means or not. The component such as borrowings (with or without interest), contribution from friends and relatives (with or without repaying option) and sale of assets is combined defined as distressed financing [27, 46].

### Indicators of socioeconomic status (SES)

We focused on three SES indicators: household monthly per capita expenditure (MPCE) quintile, education and social group of the cancer patient. Significant milestones of the Indian education system were followed to categorize the patients as illiterate (no formal schooling), primary education or below (1–5 years), middle school education or below (6–10 years), secondary education (11–12 years) and higher education (graduate school and above). Social group was categorized as scheduled tribes (ST), scheduled castes (SC), other backward classes (OBC) and other castes. The SC and ST households have historically been economically, socially and geographically deprived groups in India whereas the ‘other castes’ households have, on average, relatively better SES compared to the SC and ST households. In addition, we also include information on household location (urban vs. rural), sex of the patient, religion (Hindu, Muslim, Christian, or other) and region of residence.

### Statistical analyses

We report levels of cancer prevalence as well as treatment expenditure across socioeconomic categories. The concentration index (CI) is used to discern the socioeconomic gradient in cancer prevalence and its healthcare utilization [47–48] with focus on public and private hospitals separately. The value of CI ranges between +1 and -1 with zero depicting no inequality and large positive values indicating greater concentration of elderly persons among the richer households. Further, we employ multilevel logistic regression (adjusting for state and community level random effects) to understand the mutually adjusted associations of cancer prevalence with various SES factors in a multivariate framework. In addition, we have also analysed regression estimates adjusted for age and gender interaction [49–50]. The logistic regressions estimates are reported in the form of Odds Ratio (OR) along with respective 95 percent confidence interval. These odds ratios are the relative measure of effect which allows comparisons of group relative to the reference group. The analysis was carried out in Stata 12 and MLwiN (version 2.28) using the runmlwin module [51–53]. All the analysis use sampling weights as prescribed by the NSSO [54].

## Results

### Prevalence

The self-reported cancer prevalence at national level and its distribution across socioeconomic groups by rural and urban areas is presented in Table 1. Overall, the cancer prevalence is estimated to be 83 per 100,000 persons (95% CI: 73.2; 92.7) with a greater prevalence reported in urban population (110 per 100,000 persons; 95% CI: 93.3; 142.4). The burden of cancer among elderly cohort (70+) is significantly higher at 385 per 100,000 persons (95% CI: 268.4; 502.3). However, significant prevalence of cancer among reproductive age group (15 to 49 years) is also noted (62 per 100,000 persons; 95% CI: 50.7; 73.9). Elderly in urban areas have the highest cancer prevalence (727 per 100,000 persons; 95% CI: 492.3, 962.7). Overall, cancer is more prevalent among females (96 per 100,000 persons; 95% CI: 80.7; 110.5) than males. In addition, the cancer incidence among reproductive age group (15 to 49 years) is three times higher in females (96 per 100,000 persons; 95% CI: 76.2; 117.1) than males (30 per 100,000 persons; 95% CI: 18.2; 40.7). The prevalence among illiterates is estimated to be 79 per 100,000 persons (95% CI: 61.7; 96.0). A significant gradient in cancer prevalence can be observed across MPCE quintiles with the disease being more prevalent in high-income households (110; 95% CI: 85.6; 135.1 and 147; 95% CI: 116.8; 176.7 per 100,000 persons in fourth and fifth MPCE quintiles respectively). The gradient across MPCE quintiles is further confirmed through the concentration index of inpatient (Index value: 0.299; SE: 0.040) and outpatient (Index value: 0.322; SE: 0.496) care cases (S1 Table). Besides, the incidence of cancer displays huge inter-state variations with high prevalence across south Indian states (S2 Table).

**Table 1:** Cancer prevalence per 100,000 persons by background characteristics and place of residence, India, National Sample Survey, 2014.

Background characteristics	All India		Rural India		Urban India	
	Prevalence	95% CI	Prevalence	95% CI	Prevalence	95% CI
Age						
0–14 years	16	[7.9, 23.5]	14	[4.7, 24.1]	19	[5.7, 33.2]
15–49 years	62	[50.7, 73.9]	60	[45.1, 75.7]	66	[48.5, 84.4]
50–59 years	192	[142.5, 240.9]	158	[98.2, 217.2]	268	[180.1, 355.9]
60–69 years	321	[237.5, 404.6]	289	[182.7, 394.8]	391	[252.6, 530.4]
70+ years	385	[268.4, 502.3]	231	[107.2, 355.4]	727	[492.3, 962.7]
Sex						
Male	71	[58.4, 83.7]	56	[40.9, 70.6]	106	[82.8, 130.0]
Female	96	[80.7, 110.5]	88	[68.6, 106.4]	115	[89.9, 139.7]
Reproductive Age and Sex						
Male: 15 to 49 years	29	[18.2, 40.7]	24	[10.5, 38.1]	40	[20.6, 59.9]
Female: 15 to 49 years	96	[76.2, 117.1]	97	[69.9, 124.9]	95	[64.5, 125.5]
Education						
Illiterate	79	[61.7, 96.0]	75	[54.8, 96.1]	93	[60.8, 124.4]
Primary	53	[38.6, 68.1]	47	[29.4, 64.8]	71	[43.1, 97.9]
Secondary	48	[33.2, 63.4]	38	[20.3, 56.7]	68	[41.6, 95.1]
Higher	72	[49.7, 94.7]	36	[9.6, 63.6]	102	[69.1, 135.7]
MPCE quintile						
Lowest	49	[33.0, 65.3]	33	[15.0, 51.0]	92	[59.1, 124.4]
Second	51	[34.0, 68.1]	40	[19.5, 61.2]	75	[45.1, 105.5]
Third	61	[42.1, 80.4]	37	[16.4, 57.1]	112	[73.9, 149.8]
Fourth	110	[85.6, 135.1]	107	[76.3, 137.9]	119	[76.6, 160.3]
Highest	147	[116.8, 176.7]	143	[104.9, 180.2]	156	[105.7, 205.5]
Social group						
Scheduled tribe	42	[22.5, 60.9]	27	[9.4, 45.2]	158	[82.5, 233.9]
Scheduled caste	81	[57.1, 104.2]	75	[46.8, 103.9]	99	[56.1, 142.6]
Other backward classes	89	[73.1, 105.1]	82	[61.5, 101.9]	107	[80.1, 1333.3]
Others	89	[70.4, 107.1]	69	[45.4, 93.7]	114	[86.3, 142.4]
All India	83	[73.2, 92.7]	71	[59.3, 83.2]	110	[93.3, 127.6]
Age-Standardized Prevalence	97	[53.2, 146.1]	83	[28.0, 141.9]	130	[46.0, 211.4]

Source: Computed by Author using data from NSS 71^st^ health round

Note: Standard Population Distribution from World Health Organization (Ahmad et al 2001) is used for computing Age-Standardized Prevalence.

We use multilevel logistic regression to examine the association between socioeconomic correlates and cancer prevalence (Table 2). Age of an individual is strongly associated with cancer and elderly cohort has almost five-time higher prevalence (OR: 4.81, 95% CI: 3.50; 6.62) than the young population aged 15–24 years. In addition, the probability of cancer prevalence among reproductive age cohort (15 to 49 years) is higher for females (OR: 1.10; 95% CI: 0.86; 1.33). The odds of cancer prevalence among individuals from highest MPCE quintiles are 3.8 times that of those from the lowest MPCE quintile. The adjusted regression estimates reveal a significant gradient across educational categories whereby individuals with higher education display lower risk of cancer (OR 0.17, 95% CI: 0.13; 0.22). Therefore, reciprocal adjustment is critical to confirm the simple cross-tabulation based association and suggests that in India for a given age and income level, an individual having lower education is at a higher risk of cancer.

**Table 2:** Multilevel logistic regression estimates regarding association of socio-economic background with cancer prevalence and cancer hospitalization in private sector in India, NSS 2014.

Background characteristics	Cancer Prevalence (IPD or OPD)		Cancer: Private Hospitalization	
	OR	95% CI	OR	95% CI
Age 15–24®	1.00	-	1.00	
Age (0to5)	0.06***	[0.03, 0.14]	3.19	[0.53, 19.0]
Age (6to14)	0.71	[0.44, 1.14]	0.87	[0.30, 2.53]
Age (25to59)	2.80***	[2.07, 3.78]	1.36	[0.66, 2.82]
Age (60+)	4.82***	[3.43, 6.78]	0.94	[0.43, 2.03]
Male–Age15to 49®	1.00	-	-	-
Female–Age15to49	1.10**	[0.86, 1.33]	-	-
Female®	1.00		1.00	
Male	1.11	[0.93, 1.32]	0.89	[0.64, 1.23]
Illiterate®	1.00		1.00	
Primary Education	0.31***	[0.25, 0.38]	0.73	[0.47, 1.14]
Secondary Education	0.22***	[0.18, 0.27]	1.56*	[0.94, 2.57]
Higher Education	0.17***	[0.13, 0.22]	2.18**	[1.18, 4.02]
Casual Labour®	1.00		1.00	
Self-employed (Agriculture)	1.01	[0.79, 1.27]	1.39	[0.82, 2.36]
Self-Employed	1.10	[0.88, 1.38]	2.10***	[1.24, 3.56]
Regular Salaried	1.18	[0.93, 1.50]	1.86**	[1.10, 3.16]
Others	1.32*	[0.97, 1.80]	1.55	[0.77, 3.09]
Rural®	1.00		1.00	
Urban	1.64***	[1.37, 1.97]	1.09	[0.72, 1.63]
Lowest Income Quintile®	1.00	]	1.00	
Second Income Quintile	1.43***	[1.10, 1.86]	1.09	[0.60, 1.98]
Third Income Quintile	1.69***	[1.30, 2.20]	1.45	[0.79, 2.64]
Fourth Income Quintile	2.29***	[1.78, 2.96]	2.08**	[1.16, 3.71]
Highest Income Quintile	3.77***	[2.91, 4.88]	2.17***	[1.21, 3.91]
Hindu®	1.00		1.00	
Muslim	0.94	[0.76, 1.17]	0.76	[0.46, 1.23]
Other Religion	0.98	[0.72, 1.34]	1.12	[0.54, 2.32]
Schedule Tribes®	1.00	]	1.00	
Schedule Castes	1.16	[0.83, 1.61]	1.03	[0.48, 2.18]
Other Backward Classes	1.21	[0.90, 1.64]	1.33	[0.67, 2.66]
Other Social Group	1.41***	[1.04, 1.91]	1.59	[0.79, 3.18]
Central India®	1.00		1.00	
North India	0.89	[0.54, 1.48]	0.24***	[0.09, 0.59]
East India	1.25	[0.73, 2.14]	0.53	[0.20, 1.38]
North-East India	1.44	[0.85, 2.44]	0.28**	[0.10, 0.74]
South India	1.06	[063, 1.77]	0.87	[0.34, 2.21]
West India	0.66	[0.36, 1.22]	0.93	[0.30, 2.89]
N	335499		806	

Note

*p < .05.

**p < .01.

***p < .001.

® refers to the reference category of the variables. ORs obtained from multilevel logistic regression adjusting for community- and state-level fixed effects. The models include an intercept term.

Further, about 61 percent of cancer inpatient cases are utilizing private facilities in urban areas (S3 Table). Female patients have greater use of private hospitals for inpatient care and it is even higher (76 percent) for those from reproductive ages. Besides, the educated sections also report greater utilization of private sector services (about 75%). The regression estimates further substantiates the observed socioeconomic gradient in healthcare utilization (Table 2). For instance, the odds of seeking private hospitalization are higher for patients from richer households i.e. fourth (OR: 2.08; 95% CI: 1.16; 3.71) and fifth (OR: 2.17; 95% CI: 1.21; 3.91) MPCE quintile. In addition to this, the probability of getting private inpatient care is significantly higher for households with self-employed (OR: 2.10; 95% CI: 1.24; 3.56) and regular salaried (OR: 1.86; 95% CI: 1.10; 3.16) members.

### Out of pocket expenditure

Table 3 reports the average OOP (medical and total) expenditure and distressed financing for cancer inpatient care in public and private sector separately. The average total expenditure is estimated to be Rs 29,066 (US $ 1715.82 at 2014 Purchasing Power Parity) and Rs. 84,320 (US $ 4977.56) for the public and the private sectors, respectively. The total expenditure for males (Rs. 27427) and females (Rs. 30835) is more or less similar in public sector, whereas, expenditure incurred on males is significantly higher in private facilities. As expected, OOP expenditure is much higher for richer households. For instance, total expenditure for highest income quintile (Rs. 95422) is more than twice the average expenditure reported by the poorest quintile (Rs. 44500). The overall medical expenditure is estimated to be around 80 to 90 percent of total expenditure on cancer inpatient treatment. The non-medical expenditure also emerges as a significant component of total cancer care expenditure.

**Table 3:** Average OOP hospitalization expenditure per cancer patient by background characteristics and public and private sector treatment, India National Sample Survey 2014.

Background characteristics	Average Hospitalization Expenditure			
	Public sector		Private sector	
	Medical	Total	Medical	Total
Age				
0–5 years	19805	30041	55136	61096
6–14 years	32391	36577	56102	67044
15–24 years	18083	20947	97068	100445
25–59 years	31084	36665	85441	91156
60+ years	16758	19912	65060	71936
Sex				
Male	22782	27427	101194	108062
Female	26448	30835	64562	70235
Education				
Illiterate	17641	23176	51754	57130
Primary	20495	24760	88644	93358
Secondary	20057	23413	37718	41202
Higher	37331	42232	121714	133020
MPCE quintile				
Lowest	-	-	-	-
Second	22064	27308	44500	48083
Third	21667	24226	44948	48857
Fourth	23117	27138	83933	92169
Highest	28645	34638	89809	95422
Social group				
Scheduled tribe	8596	10941	103079	108338
Scheduled caste	24306	27977	48389	53502
Other backward classes	23710	29528	74766	80430
Others	29994	34015	94923	103361
Place of residence				
Rural	26897	32202	72654	77903
Urban	20686	24044	86941	94443
All India	24523	29066	78045	84320

Source: Computed by Author using data from NSS 71^st^ health round

Note: Distressed financing includes borrowings, sale of assets and contribution from friends and relatives as first major source. Average Hospitalization expenditure for lowest MPCE quintile is dropped due to missing data

### Financial hardships for cancer inpatient care

#### Distressed financing

**Fig 1:**
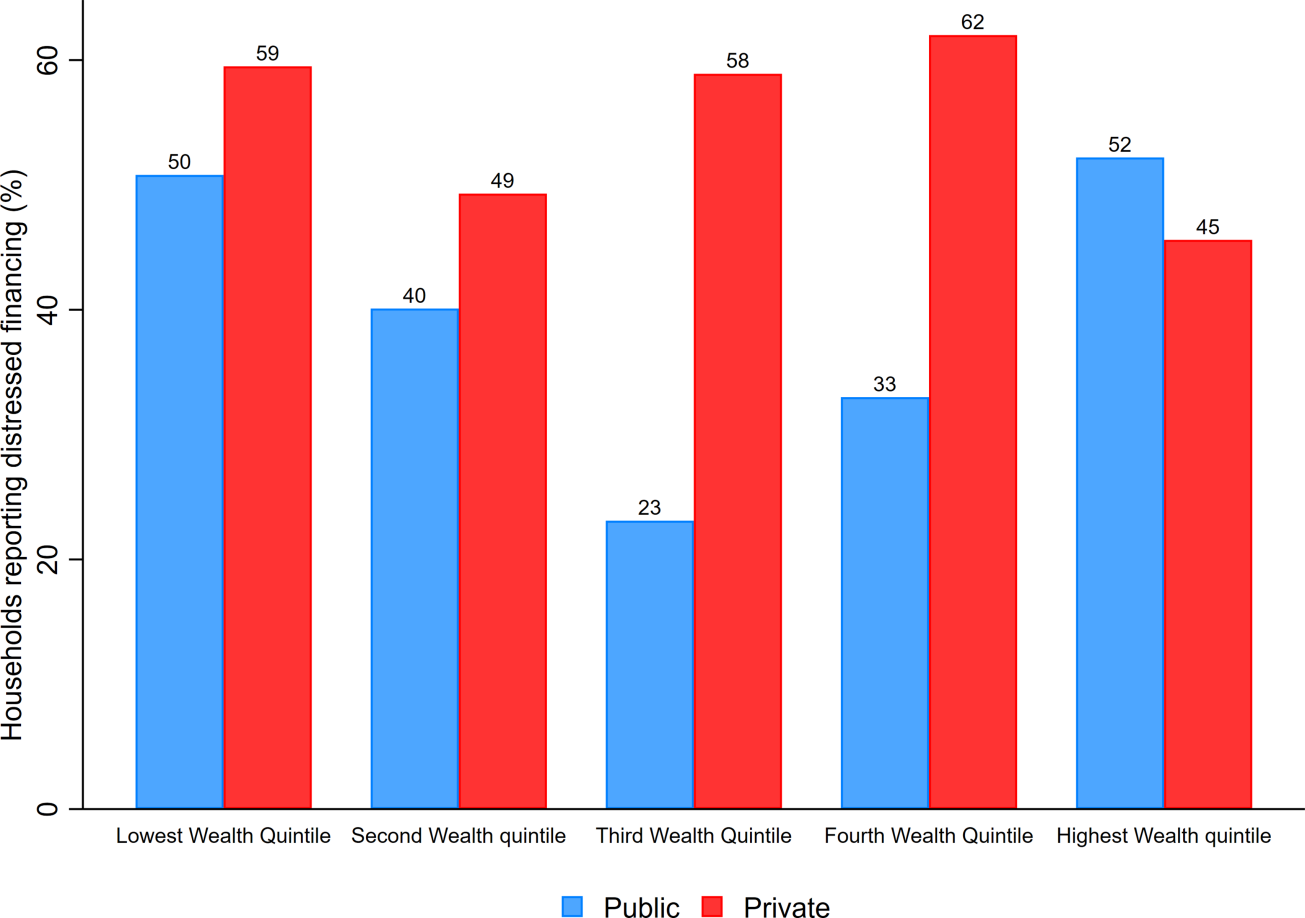
Percentage of cancer patient households reporting use of distressed financing as a major source, by wealth quintiles and public and private sector treatment, India, 2014.

The incidence of distressed financing is significantly high for both public and private hospitals across all wealth quintiles (Fig 1). About 50 percent of low-income households raise greater proportion of the cancer treatment expenditure via such distressed means. Clearly, the incidence of distressed financing is more visible among those getting treatment in private facilities across all socio-economic groups except for highest MPCE quintile. Although no clear socioeconomic gradient is observed, but the incidence of resorting to distressed means is substantially higher among poor households. At national level, more than 40 percent household resort to distress means as the main source of financing cancer treatment in public hospitals (S4 Table). A higher proportion of households (50 percent) endure such financial hardships while seeking cancer treatment in private hospitals (S4 Table). There is an overwhelming incidence of distressed financing in rural areas with 48.7and 58.4 percent of households using such means for treatment in public and private hospitals, respectively (S4 Table).

#### Catastrophic out of pocket expenditure

Finally, it is observed that a significant proportion of cancer patient households spend more than 10, 20 and 40 percent of their annual per capita household expenditure on inpatient treatment (Table 4). Overall, about 36.3 and 33.7 percent of households with cancer patients are spending more than 10 percent of their annual per capita household expenditure on public and private healthcare facilities, respectively. The incidence of catastrophic expenditure is highly concentrated among poor households. For instance, more than 50 percent patients from low income households are reported to be spending more than 10 and 20 percent of per capita household expenditure whereas, about 26 percent of richer households are reported to be spending more than 10 and 20 percent of their annual income. The catastrophic effects are substantially high for those seeking treatment in private hospitals (Table 4).

**Table 4:** Percentage of households incurring catastrophic expenditure (> 10, >20 and >40 percent of annual per capita household expenditure) on cancer hospitalization by demographic and socioeconomic background characteristics, India: NSS 2014.

Background characteristics	Public Sector			Private sector		
	At 10%	At 20%	At 40%	At 10%	At 20%	At 40%
Age						
0–5 years	46.2	46.2	46.2	87.3	87.3	87.3
6–14 years	57.9	50.5	48.9	42.5	42.5	41.8
15–24 years	31.6	30.5	25.8	68.1	62.4	59.6
25–59 years	33.6	32.2	27.2	67.2	65.3	63.3
60+ years	39.3	34.6	26.9	57.9	55.0	58.8
Sex						
Male	42.7	39.9	32.7	56.9	55.3	53.1
Female	31.6	29.1	24.6	68.9	66.2	61.9
Education						
Illiterate	33.2	30.8	26.7	66.4	65.5	61.9
Primary	31.6	29.9	25.2	63.5	61.4	57.9
Secondary	32.8	29.8	23.5	68.2	63.2	59.1
Higher	23.4	22.9	22.5	76.8	74.2	72.4
MPCE quintile						
Lowest	55.9	52.1	44.7	42.3	42.3	42.3
Second	48.6	43.8	32.9	57.2	53.5	52.7
Third	38.5	35.2	22.1	55.9	55.6	53.9
Fourth	31.5	30.3	29.8	70.5	70.5	66.6
Highest	28.9	26.6	22.3	70.2	65.2	59.8
Social group						
Scheduled tribe	66.6	64.3	33.3	45.2	45.2	40.1
Scheduled caste	38.4	36.8	30.1	65.0	59.4	56.7
Other backward classes	31.1	26.9	24.5	64.8	62.4	59.7
Others	37.2	36.1	30.9	64.9	64.9	60.4
Place of residence						
Rural	36.5	33.9	30.5	64.8	62.1	58.7
Urban	35.9	33.1	23.4	62.1	60.6	57.4
Religion						
Hindu	35.8	33.2	27.3	65.2	62.5	59.1
Muslim	39.4	37.0	33.4	55.7	55.7	51.4
Other religion	36.9	33.6	27.5	59.7	59.7	59.7
All India	36.3	33.7	28.0	63.8	61.6	58.2

Source: Computed by author using NSS 71^st^ round.

Note: The health expenditure is said to be ‘catastrophic’ when it exceeds a certain proportion (in this table: 10, 20 and 40 percent) of household income or ability to pay (Berki 1986; Xu et al. 2003)

The present analysis, however, is sensitive to the following limitations. First, the cross-sectional nature of data and survey design does not allow us to infer causality. Second, the results presented here are based on self-reported household survey information. Due to data limitations, we are also not able to assess the respective contributions of different types of sources of financing in total out of pocket expenditure. Similarly, a lack of information on the nature of cancer further prohibits any analysis associated with these dimensions. Finally, because of sample size limitations we could not assess the socioeconomic patterns across all regions and states in India.

## Discussion and conclusion

Increasing prevalence of cancer is a major public health concern. The issue assumes utmost relevance for developing countries such as India because of large population base, limited diagnostic facilities, very high treatment costs and poor survival prospects. Given the nature and consequences of the ailment, it is therefore critical to systematically track the distributional and financial aspects of the disease through rigorous nationwide assessments. In this regard, we find that the overall self-reported prevalence of cancer is estimated to be 83 per 100,000 persons with a greater prevalence in urban areas (110 per 100,000 persons). The figures correspond with the overall cancer mortality estimates presented by World Health Organization (i.e. 75 per 100000 persons) [18]. In addition to this, the age-standardized rate for cancer prevalence is estimated to be 97 per 100,000 persons. These estimates are also similar to the age-adjusted cancer incidence (94 per 100,000 persons) discussed in [55].

The higher burden of cancer among elderly cohort and in demographically advanced states implies greater requirements of tertiary care facilities in these regions. The cancer incidence among reproductive age cohort is considerably high in females. These estimates call for a greater policy emphasis on higher burden of breast (27 percent of all cancer cases in females) and uterine cancer among females [56–58]. Further a strong income gradient in cancer prevalence reflects higher incidence of cancer among richer households. It is however important to understand that the treatment seeking may be higher among richer households and therefore the cancer cases among poor may be underreported. Further, the access to cancer diagnosis and screening facilities are better equipped and agglomerated in urban settings due to which richer section reveals higher prevalence. Besides, regression estimates reflect significantly higher odds of cancer prevalence among illiterates as compared to any other educational group. These patterns warrant further understanding of the nature of cancer prevalence and variations across education and income groups.

Private sector health care facilities are more accessed for cancer treatment in India. In particular, richer households rely more on private hospitals for cancer inpatient care, whereas poor households mainly depend on public healthcare facilities. Further the average OOP expenditure is much higher for richer households as compared to poor households. Besides, average expenditure in private facilities is almost two times higher than public facilities. We also find that, treatment of cancer causes substantial financial shocks and affect the usual living standard of households. Evidently, one in every three cancer patient’s households spends about half of per capita annual household expenditure on cancer hospitalization. The prevalence of catastrophic expenditure is highly concentrated among poor households implying great importance of wealth and physical capital in ensuring quality tertiary healthcare. A large number of households in low-income countries incur financial debts and sell assets in order to finance their health care payments especially for NCDs like CVD and cancer [25, 46, 59]. In this context, we find that more than 50 percent of low-income households depend on distress means as major source of financing for cancer hospitalization. Further, rural households have higher tendency to depend on distress means to finance the healthcare payments. This is perhaps because poorer households are more deprived and thus resort to borrowings at first place. Importantly, almost all households with cancer patients resort to distress means of health care financing.

Given the magnitude and financial implications, a two-pronged policy approach is essential. First, it is critical to target the risk factors of cancer prevalence across all the sub sections of population and second, to ensure quality and affordable care to all cancer patients. It is widely discussed that India is experiencing accelerated aging; therefore, it is desirable to develop special geriatric oncology facilities as elder cancer patients–in most cases–cannot sail through the general treatment and therapies [60]. Further, studies also suggest that oncology research for older patients must include comprehensive clinical geriatric assessments, improved biological assessments, more trials tailored for oldest old cohort (75+ years) and enhanced infrastructure [61–63].

High prevalence of cancer among reproductive age cohort, particularly in females, cautions significant policy attention along with specific efforts for pediatric cancer treatment that currently lacks priority in policy discourse [63]. In this regard, it is worth mentioning that cervical cancer mainly caused by sexually transmitted *Human Papilloma virus* (HPV) is ranked as most frequent form of cancer among Indian women [64]. Studies have suggested that about three fourth of sexually active adults are likely to be affected by any one type of HPV [65]. In light of this, the HPV vaccination is of public health relevance, but Indian academy of Pediatrics and Committee on Immunization (IAPCOI) recommends offering vaccines to only those who can afford. It is important to understand that vaccines are effective only prior to infection, therefore it is necessary to provide the vaccination before sexual debut. The observed gradient across SES factors significantly reflects the importance of lifestyle factors in cancer prevalence. Policies should aim to curb universal risk factors causing cancerous tumors such as tobacco and alcohol, poor diet (insufficient fruit or vegetable intake), overweight and obesity, physical inactivity, chronic infections from Hepatitis B and C virus and environmental risks including ionizing and non-ionizing radiation [5, 8,66–68]. These issues are critical because India has high prevalence of such risk factors. For instance, India has third highest increase in alcohol per capita (APC) consumption between 1992 and 2012 among 40 countries [65]. In addition to this, India has the third highest number of obese individuals in the world after USA and China [69–70].

A few studies suggest that more than half of the cancer cases can be successfully treated if detected at right stage [71]. Therefore, it is critical to ensure improved rates of cancer survivorship through prevention, early detection, diagnosis and treatment. The detection rate in India, however, is very low and about only 20 to 30 percent of cases is diagnosed at stage I and II, respectively. This calls for increasing general awareness regarding cancer symptoms, causes, preventive measures and treatment options. Although, National Cancer Control Programme (NCCP) was formulated in 1984 with four major goals i.e. primary prevention of tobacco related cancers, early detection of cancers, augmentation of treatment facilities and establishing palliative care. But certainly, there are no international standards of practices for early detection of oral cancers despite the fact that most of the oral cancers are found in South-Asian countries [40]. Though National Cancer Registry Programme (1982) have been providing authentic information on cancer incidence since more than 30 years, but the functioning of NCRP is based on just 28 Population Based Cancer Registries (PBCRs).

These findings clearly outline the need for greater public health investments in cancer treatment facilities including infrastructure, medical practitioners and accessibility. For instance, with the given population of country, there is requirement of 1200 radiotherapy machines, whereas only 400 machines are available at present for cancer treatment [24]. Besides most of the modern cancer treatment facilities are concentrated in private hospitals which are extremely expensive. For example, a single course of radiotherapy in private hospitals costs around Rs. 117000. Evidently this does not include the expenses on further treatment like surgery, chemotherapy and other supportive medicines. In addition to this, higher non-medical expenditure for rural households suggests urgent need of expanding cancer treatment facilities in backward and rural areas. Such high catastrophic spending and distressed financing further emphasizes on the need for quality and affordable cancer treatment. The findings of suggest that financial catastrophe of cancer inpatient treatment is very high, therefore insurance cover for cancer treatment is equally desirable in government insurance policies such as *Pradhan Mantri Suraksha Bima Yojana* (PMSBY), particularly for vulnerable and destitute households.

Following such impoverishing and catastrophic effects, there needs to be more clarity from policies regarding financing mechanisms for such high-cost disease. The relevance of such policy guidelines is all the more necessary because of a prominent role envisaged by the National Health Policy 2017 the private sector in secondary and tertiary health care [45]. Thus, in concluding, it is important to reiterate that cancer treatment in India should be received as a priority both to improve cancer survival and to protect households from financial catastrophe.

At this point, it is important to mention that this study is based on National Sample Survey (NSS) self-reported data on cancer. While this study mainly aims at analyzing out of pocket expenditure and financial hardships on cancer inpatient treatment, information on availability and cost of drugs, access to modern techniques of treatment is also desirable. Further, estimates on catastrophic expenditure at different thresholds (i.e. 10%, 20% and 30%) across different population groups does-not reveal the information about willingness of households to spend on cancer care. However, it will be interesting to explore the household level differences in willingness to spend on healthcare in general and chronic diseases in particular.

## Supporting information

S1 Table Concentration index values for distribution of cancer infected persons, cancer hospitalization and cancer ailments in last 15 days across monthly per capita consumption expenditure, India National Sample Survey, 2014. (DOCX)

S2 Table Cancer: Inpatient and outpatient cases reported per 100,000 persons by state of residence, India National Sample Survey, 2014. (DOCX)

S3 Table Number of inpatient and outpatient care cases of cancer and percentage distribution by sector of treatment by background characteristics, India, National Sample Survey, 2014. (DOCX)

S4 Table Percentage of cancer patient households reporting use of distressed financing as a major source, by background characteristics and public and private sector treatment, India National Sample Survey 2014. (DOCX)

S5 Table Calculation of Age Standardized Rate (Mean) of cancer prevalence in India, National Sample Survey, 2014. (DOCX)

S6 Table Calculation of Age Standardized Rate (Mean) of cancer prevalence in Rural India, National Sample Survey, 2014. (DOCX)

S7 Table Calculation of Age Standardized Rate (Mean) of cancer prevalence in Urban India, National Sample Survey, 2014. (DOCX)
