# Supplementary material for: Economic burden of cancer in India: Evidence from cross-sectional nationally representative household survey, 2014
Source: PLoS One. 2018 Feb 26;13(2):e0193320. doi: 10.1371/journal.pone.0193320 (PMC5826535; doi:10.1371/journal.pone.0193320)
Supplement: S1 Table — (DOCX) [file pone.0193320.s001.docx]

**Table S1:** Concentration index values for distribution of cancer infected persons, cancer hospitalization and cancer ailments in last 15 days across monthly per capita consumption expenditure, India National Sample Survey, 2014

| Concentration index | Cancer diagnosed Persons | Cancer Hospitalization | Cancer ailment in last 15 days |
| --- | --- | --- | --- |
| Rural India | 0.330*** | 0.340*** | 0.393*** |
| *Std. error* | *[0.049]* | *[0.055]* | *[0.073]* |
| Urban India | 0.152*** | 0.203*** | 0.160 |
| *Std. error* | *[0.046]* | *[0.056]* | *[0.629]* |
| All India | 0.287*** | 0.299*** | 0.322*** |
| *Std. error* | *[0.034]* | *[0.040]* | *[0.496]* |

Note: *** and ** denotes significance at 1 percent and 5 percent level, respectively.

Source: Computed by Author using data from NSS 71^st^ health round
