# Supplementary material for: Economic burden of cancer in India: Evidence from cross-sectional nationally representative household survey, 2014
Source: PLoS One. 2018 Feb 26;13(2):e0193320. doi: 10.1371/journal.pone.0193320 (PMC5826535; doi:10.1371/journal.pone.0193320)
Supplement: S2 Table — (DOCX) [file pone.0193320.s002.docx]

**Table S2:** Cancer: Inpatient and Outpatient cases reported per 100,000 persons by state of residence, India National Sample Survey, 2014

| States | Inpatient Care | | | Outpatient Care | | |
| --- | --- | --- | --- | --- | --- | --- |
|  | All | Public | Private | All | Public | Private |
| Andhra Pradesh | 189 | 11 | 178 | 97 | 0 | 97 |
| Assam | 27 | 17 | 10 | - | - | - |
| Bihar | 18 | 6 | 12 | 4 | - | 4 |
| Delhi | 81 | 48 | 33 | 40 | 34 | 66 |
| Gujarat | 73 | 45 | 28 | 14 | 5 | 8 |
| Karnataka | 120 | 31 | 89 | 23 | 10 | 12 |
| Kerala | 403 | 263 | 140 | 110 | 74 | 36 |
| Madhya Pradesh | 55 | 21 | 34 | 37 | 24 | 9 |
| Maharashtra | 70 | 9 | 61 | 34 | 4 | 29 |
| Odisha | 109 | 59 | 50 | 31 | 21 | 10 |
| Punjab | 64 | 5 | 59 | 28 | 3 | 25 |
| Rajasthan | 62 | 36 | 26 | 49 | 24 | 6 |
| Tamil Nadu | 115 | 45 | 70 | 124 | 54 | 69 |
| Uttar Pradesh | 72 | 25 | 47 | 41 | 9 | 25 |
| West Bengal | 107 | 56 | 51 | 40 | 14 | 25 |

Source: Computed by Author using data from NSS 71^st^ health round

Note: Sum of Public and Private may not be 100 as some cases were reported to be missing/not treated
