# Supplementary material for: Economic burden of cancer in India: Evidence from cross-sectional nationally representative household survey, 2014
Source: PLoS One. 2018 Feb 26;13(2):e0193320. doi: 10.1371/journal.pone.0193320 (PMC5826535; doi:10.1371/journal.pone.0193320)
Supplement: S3 Table — (DOCX) [file pone.0193320.s003.docx]

**Table S3:** Number of inpatient and outpatient care cases of cancer and percentage distribution by sector of treatment by background characteristics, India, National Sample Survey, 2014

| Background characteristics | Inpatient per 100,000 persons | | | Outpatient cases per 100,000 persons | | |
| --- | --- | --- | --- | --- | --- | --- |
|  | All Cases | Public (%) | Private (%) | All Cases | Public (%) | Private (%) |
| Age |  |  |  |  |  |  |
| 0 - 5 years | 16 | 46 | 54 | 17 | 0 | 100 |
| 6 – 14 years | 15 | 49 | 51 | 14 | 57 | 21 |
| 15 – 24 years | 17 | 34 | 66 | 11 | 91 | 9 |
| 25 – 59 years | 108 | 35 | 65 | 53 | 40 | 51 |
| 60+ years | 400 | 46 | 54 | 151 | 25 | 72 |
| Sex |  |  |  |  |  |  |
| Male | 73 | 48 | 52 | 33 | 27 | 67 |
| Female | 102 | 33 | 67 | 48 | 40 | 52 |
| Reproductive Age*Sex |  |  |  |  |  |  |
| Male: 15 to 49 years | 29 | 48 | 52 | 11 | 36 | 74 |
| Female: 15 to 49 years | 90 | 24 | 76 | 53 | 43 | 57 |
| Education |  |  |  |  |  |  |
| Illiterate | 67 | 37 | 63 | 48 | 33 | 58 |
| Primary | 57 | 44 | 56 | 35 | 37 | 51 |
| Secondary | 34 | 29 | 71 | 29 | 59 | 41 |
| Higher | 63 | 25 | 75 | 49 | 12 | 88 |
| MPCE quintile |  |  |  |  |  |  |
| Lowest | 34 | 58 | 42 | 27 | 52 | 26 |
| Second | 55 | 45 | 55 | 16 | 38 | 50 |
| Middle | 64 | 45 | 55 | 18 | 44 | 22 |
| Fourth | 109 | 38 | 62 | 66 | 17 | 82 |
| Highest | 183 | 33 | 67 | 74 | 43 | 55 |
| Social group |  |  |  |  |  |  |
| Scheduled tribe | 58 | 53 | 47 | 10 | 20 | 60 |
| Scheduled caste | 87 | 43 | 57 | 42 | 26 | 74 |
| Other backward classes | 86 | 37 | 63 | 44 | 43 | 50 |
| Others | 100 | 38 | 62 | 43 | 28 | 60 |
| Place of residence |  |  |  |  |  |  |
| Rural | 77 | 40 | 60 | 33 | 39 | 58 |
| Urban | 112 | 39 | 61 | 58 | 29 | 60 |
| All India | 87 | 40% | 60% | 40 | 35% | 58% |

Source: Computed by Author using data from NSS 71^st^ round, 2014

Note: Sum of Public and Private may not be 100 as some cases were reported to be missing/not treated
