# Supplementary material for: Economic burden of cancer in India: Evidence from cross-sectional nationally representative household survey, 2014
Source: PLoS One. 2018 Feb 26;13(2):e0193320. doi: 10.1371/journal.pone.0193320 (PMC5826535; doi:10.1371/journal.pone.0193320)
Supplement: S5 Table — (DOCX) [file pone.0193320.s005.docx]

**Table S5:** Calculation of Age Standardized Rate (Mean) of Cancer Prevalence in India, National Sample Survey, 2014

| Age Group (Years) | Population | Cancer (Nos.) | Ci | Si | Mi = Ci*Si |
| --- | --- | --- | --- | --- | --- |
| 0-4 | 29758 | 3 | 0.000101 | 0.0886 | 0.0000089 |
| 5-9 | 32189 | 7 | 0.000217 | 0.0869 | 0.0000188 |
| 10-14 | 35152 | 5 | 0.000142 | 0.086 | 0.0000122 |
| 15-19 | 32273 | 7 | 0.000217 | 0.0847 | 0.0000183 |
| 20-24 | 30135 | 6 | 0.000199 | 0.0822 | 0.0000163 |
| 25-29 | 28062 | 5 | 0.000178 | 0.0793 | 0.0000141 |
| 30-34 | 25749 | 13 | 0.000505 | 0.0761 | 0.0000384 |
| 35-39 | 25201 | 37 | 0.001468 | 0.0715 | 0.0001049 |
| 40-44 | 21497 | 23 | 0.00107 | 0.0659 | 0.0000705 |
| 45-49 | 18381 | 21 | 0.001142 | 0.0604 | 0.0000690 |
| 50-54 | 15147 | 30 | 0.001981 | 0.0537 | 0.0001063 |
| 55-59 | 15247 | 28 | 0.001836 | 0.0455 | 0.0000835 |
| 60-64 | 9525 | 34 | 0.00357 | 0.0372 | 0.0001327 |
| 65-69 | 7539 | 20 | 0.002653 | 0.0296 | 0.0000785 |
| 70-74 | 4989 | 14 | 0.002806 | 0.0221 | 0.0000620 |
| 75-79 | 2335 | 10 | 0.004283 | 0.0152 | 0.0000650 |
| 80-84 | 1361 | 4 | 0.002939 | 0.0091 | 0.0000267 |
| 85+ | 957 | 7 | 0.007315 | 0.0063 | 0.0000460 |
|  |  |  |  |  | **0.000973** |

Source: Computed by Author using data from NSS 71^st^ round, 2014

Note: Ci = Cancer Cases / Population in that age group, Si – Standard Distribution of Population by World Bank (Ahmad et al. 2001)
