# Supplementary material for: Economic burden of cancer in India: Evidence from cross-sectional nationally representative household survey, 2014
Source: PLoS One. 2018 Feb 26;13(2):e0193320. doi: 10.1371/journal.pone.0193320 (PMC5826535; doi:10.1371/journal.pone.0193320)
Supplement: S6 Table — (DOCX) [file pone.0193320.s006.docx]

**Table S6:** Calculation of Age Standardized Rate (Mean) of Cancer Prevalence in Rural India, National Sample Survey, 2014

| Age Group (Years) | Population | Cancer (Nos.) | Ci | Si | Mi = Ci*Si |
| --- | --- | --- | --- | --- | --- |
| 0-4 | 17961 | 2 | 0.000111 | 0.0886 | 0.00000987 |
| 5-9 | 19452 | 5 | 0.000257 | 0.0869 | 0.00002234 |
| 10-14 | 21002 | 1 | 4.76E-05 | 0.086 | 0.00000409 |
| 15-19 | 18969 | 3 | 0.000158 | 0.0847 | 0.00001340 |
| 20-24 | 16805 | 3 | 0.000179 | 0.0822 | 0.00001467 |
| 25-29 | 15293 | 2 | 0.000131 | 0.0793 | 0.00001037 |
| 30-34 | 13692 | 3 | 0.000219 | 0.0761 | 0.00001667 |
| 35-39 | 13900 | 26 | 0.001871 | 0.0715 | 0.00013374 |
| 40-44 | 11733 | 12 | 0.001023 | 0.0659 | 0.00006740 |
| 45-49 | 10092 | 11 | 0.00109 | 0.0604 | 0.00006583 |
| 50-54 | 8394 | 16 | 0.001906 | 0.0537 | 0.00010236 |
| 55-59 | 8692 | 11 | 0.001266 | 0.0455 | 0.00005758 |
| 60-64 | 5207 | 18 | 0.003457 | 0.0372 | 0.00012860 |
| 65-69 | 4306 | 9 | 0.00209 | 0.0296 | 0.00006187 |
| 70-74 | 2859 | 4 | 0.001399 | 0.0221 | 0.00003092 |
| 75-79 | 1280 | 2 | 0.001563 | 0.0152 | 0.00002375 |
| 80-84 | 734 | 3 | 0.004087 | 0.0091 | 0.00003719 |
| 85+ | 529 | 3 | 0.005671 | 0.0063 | 0.00003573 |
|  |  |  |  |  | **0.000836382** |

Source: Computed by Author using data from NSS 71^st^ round, 2014

Note: Ci = Cancer Cases / Population in that age group, Si – Standard Distribution of Population by World Bank (Ahmad et al. 2001)
