# Supplementary material for: Economic burden of cancer in India: Evidence from cross-sectional nationally representative household survey, 2014
Source: PLoS One. 2018 Feb 26;13(2):e0193320. doi: 10.1371/journal.pone.0193320 (PMC5826535; doi:10.1371/journal.pone.0193320)
Supplement: S7 Table — (DOCX) [file pone.0193320.s007.docx]

**Table S7:** Calculation of Age Standardized Rate (Mean) of Cancer Prevalence in Urban India, National Sample Survey, 2014

| Age Group (Years) | Population | Cancer Persons | Ci | Si | Mi = Ci*Si |
| --- | --- | --- | --- | --- | --- |
| 0-4 | 11007 | 0.1 | 0.00000909 | 0.0886 | 0.00000080 |
| 5-9 | 11863 | 1 | 0.00008430 | 0.0869 | 0.00000733 |
| 10-14 | 13382 | 6 | 0.00044836 | 0.086 | 0.00003856 |
| 15-19 | 12840 | 6 | 0.00046729 | 0.0847 | 0.00003958 |
| 20-24 | 13592 | 4 | 0.00029429 | 0.0822 | 0.00002419 |
| 25-29 | 13287 | 4 | 0.00030105 | 0.0793 | 0.00002387 |
| 30-34 | 12795 | 12 | 0.00093787 | 0.0761 | 0.00007137 |
| 35-39 | 11638 | 7 | 0.00060148 | 0.0715 | 0.00004301 |
| 40-44 | 10147 | 11 | 0.00108406 | 0.0659 | 0.00007144 |
| 45-49 | 8572 | 11 | 0.00128325 | 0.0604 | 0.00007751 |
| 50-54 | 6925 | 15 | 0.00216606 | 0.0537 | 0.00011632 |
| 55-59 | 6543 | 21 | 0.00320954 | 0.0455 | 0.00014603 |
| 60-64 | 4481 | 17 | 0.00379380 | 0.0372 | 0.00014113 |
| 65-69 | 3219 | 13 | 0.00403852 | 0.0296 | 0.00011954 |
| 70-74 | 2115 | 13 | 0.00614657 | 0.0221 | 0.00013584 |
| 75-79 | 1092 | 10 | 0.00915751 | 0.0152 | 0.00013919 |
| 80-84 | 658 | 2 | 0.00303951 | 0.0091 | 0.00002766 |
| 85+ | 440 | 6 | 0.01363636 | 0.0063 | 0.00008591 |
|  |  |  |  |  | **0.0013093** |

Source: Computed by Author using data from NSS 71^st^ round, 2014

Note: Ci = Cancer Cases / Population in age group, Si – Standard Distribution of Population by World Bank (Ahmad et al. 2001)
